# Cervical Facial Actinomycosis Complicated by Bacteremia After 30 Years of Dental Neglect: Successful Treatment With Ceftriaxone and Metronidazole

**DOI:** 10.7759/cureus.51734

**Published:** 2024-01-06

**Authors:** Paul Wasuwanich, Dominique Szymkiewicz, Jaya Kumari, Diego Vanegas Acosta

**Affiliations:** 1 College of Medicine, University of Florida College of Medicine, Gainesville, USA; 2 Internal Medicine, Mehran Medical Centre, Karachi, PAK; 3 College of Medicine, University of Florida Shands Hospital, Gainesville, USA

**Keywords:** mandibular abscess, mandibular actinomycosis, dental neglect, bacteremia, actinomyces

## Abstract

Actinomycosis is an uncommon bacterial infection, caused by the Actinomyces species, and it most commonly presents as cervicofacial actinomycosis. The most common risk factors for actinomycosis are poor dental hygiene, oral surgery, maxillofacial trauma, local tissue inflammation, and diabetes. We discuss a case of a male patient in his 50s with 30 years of poor dental hygiene, complicated by tobacco use, who presented with septic shock and was found to have cervicofacial actinomycosis and bacteremia. The treatment of severe actinomycosis often involves prolonged penicillin-based antibiotic therapy. This is the first case in the literature to describe the successful treatment of cervicofacial actinomycosis and bacteremia with intravenous cefepime (later narrowed to ceftriaxone) and oral metronidazole.

## Introduction

Actinomycosis is a rare, opportunistic infection caused by Actinomyces spp., anaerobic Gram-positive bacteria that are normally found in the human mouth and gastrointestinal and genital tracts [1]. It is often misdiagnosed as it can mimic other conditions such as malignancy, tuberculosis [2], and Nocardiainfection, and a high index of clinical suspicion is required for an early diagnosis. Cervicofacial actinomycosis, often secondary to odontogenic infection, is the most frequent clinical form of actinomycosis and the most common clinical manifestation, representing approximately 60% of all reported cases [1,3]. Actinomyces species have also been reported to cause maxillary osteomyelitis in patients with odontogenic maxillary sinusitis [1]. It can also involve the respiratory tract, digestive tract, genitourinary tract, and central nervous system, but those manifestations are less frequent than cervicofacial manifestations. The initial disease manifests as coarse, inflammatory nodules, which frequently evolve into purulent, draining fistula.

Cervicofacial actinomycosis can be found worldwide with no predilection for age, race, season, or occupation [1]. The predisposing factors for cervicofacial actinomycosis are poor dental hygiene (e.g., dental caries), oral surgery (e.g., tooth extraction), maxillofacial trauma, local tissue inflammation (e.g., tonsillitis, tumor), and comorbidities such as diabetes [1].

## Case presentation

The patient was a male in his 50s with a past medical history of hypertension, tobacco use, and alcohol use disorder (approximately 20 drinks per week) who presented with a sore throat, subjective fevers, chills, and dyspnea for three days. Over those three days, his symptoms had progressively worsened and now included left neck swelling and a muffled voice. Additionally, he reported decreased appetite, dysphagia, and facial swelling. His surgical history was significant for one incision and drainage of a back abscess five years prior. Furthermore, he reportedly had never brushed his teeth or seen a dentist in the past 30 years. Physical exam was positive for poor dentition including large build-up of plaque and calculus on teeth, severe decay and fracturing of multiple teeth, and multiple missing teeth. There was left mandible and left neck swelling, warmth, and tenderness. Examination of the mouth also revealed a draining fistula intraorally near the left tonsillar region that produced serosanguinous fluid when digital pressure was applied. There was no evidence of thrush, visible bone, lacerations, or deviation of the uvula within the mouth. On presentation, he was found to be afebrile, tachycardic, and tachypneic without respiratory distress, but hypotensive to 74/54 mmHg.

Differential diagnosis and investigations

Sepsis due to a dental abscess was suspected. Initial labs revealed metabolic acidosis (pH 7.34), elevated lactic acid (5.6 mmol/L), elevated C-reactive protein (255 mg/L), and leukopenia (2.7 x 10^3^/uL) with bandemia; 11 hours later, the white blood cell count (WBC) was 18.4 x 10^3^/uL (Table 1).

**Table 1:** Laboratory investigations from admission (day 1) to discharge (day 6) Abnormal values are bolded PCR: polymerase chain reaction; SARS-CoV-2: severe acute respiratory syndrome coronavirus 2

Laboratory test	Reference values	Day 1	Day 2	Day 3	Day 6
White blood count	4.0-10.0 x 10^3^/µL	2.7	15.9	15.2	9.5
Hemoglobin	13.0-16.5 g/dL	16.2	12.3	12.7	14.5
Hematocrit	39.0-49.0%	46.9	35.2	37.0	42.0
Platelet count	150-450 x 10^3^/µL	78	42	42	194
Absolute neutrophil count	1.70-7.00 x 10^3^/µL	2.46	14.18	12.98	6.76
Absolute lymphocyte count	1.00-3.20 x 10^3^/µL	0.11	0.16	1.38	1.61
Absolute eosinophil count	0.03-0.46 x 10^3^/µL	0.00	0.00	0.09	0.19
Bands	0.0-10.0%	19.2	35.3	-	-
Sodium	136-145 mmol/L	134	137	137	137
Potassium	3.3-5.1 mmol/L	5.5	4.3	3.7	3.7
Chloride	98-107 mmol/L	102	107	104	105
Blood urea nitrogen	6-21 mg/dL	25	36	29	14
Creatinine	0.51-1.18 mg/dL	1.58	1.27	1.06	0.85
Glucose	65-99 mg/dL	112	125	101	100
Total bilirubin	0.0-1.0 mg/dL	1.9	1.1	0.6	0.6
Alkaline phosphatase	40-150 IU/L	53	49	77	83
Aspartate aminotransferase	0-37 IU/L	39	40	38	31
Alanine aminotransferase	0-50 IU/L	25	25	25	23
Blood culture, Actinomyces species	Negative	Positive	-	Negative	-
Blood culture, Prevotella species	Negative	Positive	-	Negative	-
Blood culture, Gemella bergeri	Negative	Positive	-	Negative	-
Blood culture, Streptococcus species	Negative	Positive	-	Negative	-
Nasal culture, methicillin-resistant S. aureus (MRSA)	Negative	Negative	Negative	-	-
PCR, SARS-CoV-2	Negative	Negative	-	-	-

**Figure 1:**
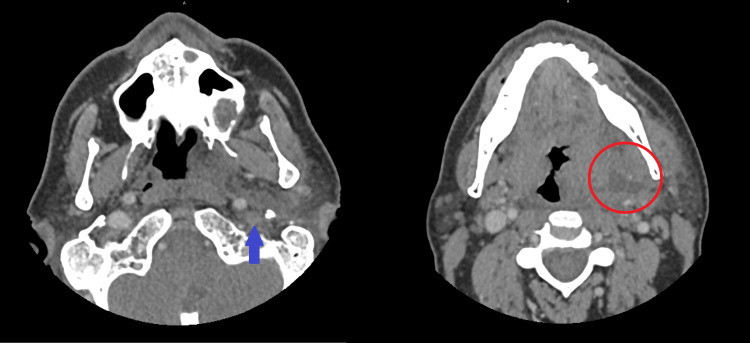
CT showing left peritonsillar abscess (red circle) and left internal jugular vein thrombosis (blue arrow) CT: computed tomography

Blood cultures were drawn concurrently. The patient then underwent a CT of the neck and maxillofacial region with intravenous contrast, which revealed a left peritonsillar abscess measuring 2.6 x 1.4 x 3.2 cm (anterior-posterior, transverse, and craniocaudal, respectively) and perforating into the parapharyngeal space as well as left internal jugular vein thrombosis concerning for Lemierre's syndrome (Figure 1).

**Figure 2:**
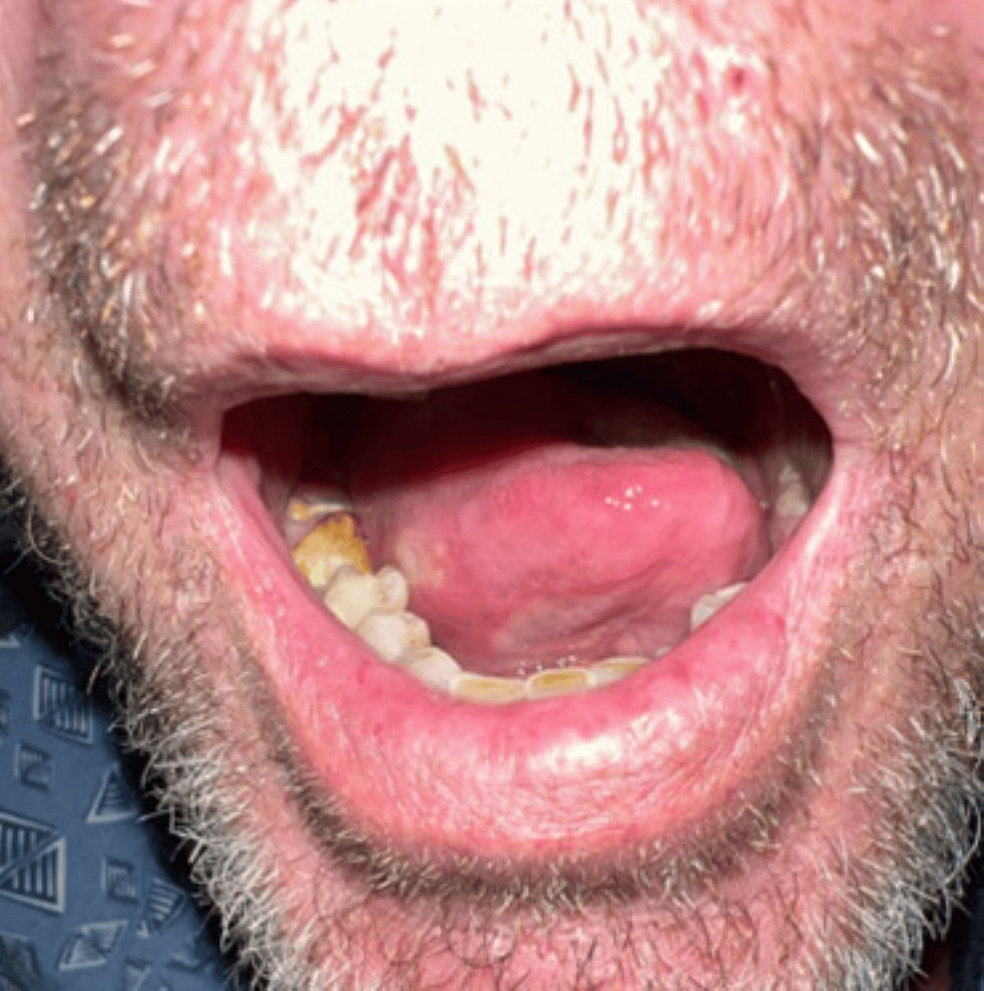
Bright yellow granules in right lower molars, which raised suspicion for Actinomyces

On admission day two (after 24 hours of admission), cultures revealed growth in both aerobic (Streptococcus species including Streptococcus pyogenes) and anaerobic blood culture bottles. Subsequent sensitivity tests revealed sensitivity to ceftriaxone (31 mm from disk diffusion test for Streptococcus pyogenes) and metronidazole. A physical exam revealed bright yellow granules in his right lower molars (Figure 2). However, mouth swabs for Actinomyces were negative.

On day three of admission, blood cultures from admission day one confirmed the presence of Streptococcus species including Streptococcus pyogenes, Prevotella species, Actinomyces species, and Gemella bergeri. Blood cultures from day three of admission showed no growth to date.

Treatment

The patient was empirically treated on admission day one with intravenous vancomycin (1500 mg once then 1000 mg to target random concentration of 10-15 mcg/mL), intravenous cefepime (2000 mg every 12 hours), intravenous clindamycin (600 mg once), and oral metronidazole (500 mg every eight hours). He was then admitted to the ICU for septic shock; his mean arterial pressure (MAP) was 63 mmHg status post four liters of Ringer’s lactate solution, but he was never started on pressors due to maintenance of MAP above 65 mmHg in the ICU. His abscess found on CT was drained by an ENT specialist, resulting in symptom improvement. 

On admission day two, after obtaining culture results, antibiotics were narrowed down to intravenous ceftriaxone (2000 mg every 24 hours) and oral metronidazole (500 mg every eight hours). The patient was then downgraded to floor status. He was discharged on day six of admission with symptom improvement and WBC levels at 9.5 x 10^3^/uL. A peripherally inserted central catheter was placed as recommended by the Infectious Disease team. Per Infectious Disease, therapy was refined to intravenous ceftriaxone 2000 mg every 24 hours and oral metronidazole 500 mg every eight hours for two weeks after the first negative blood culture. After the completion of intravenous antibiotics, the patient was sent home on amoxicillin-clavulanic acid twice a day for an additional two weeks of therapy.

## Discussion

Similar to our case, most presentations of actinomycosis are cervicofacial and the most common portal of entry of the bacteria is periodontal disease [1]. Apart from poor oral hygiene, the other predisposing factors for developing actinomycosis include the male gender, diabetes mellitus, immunosuppression, alcoholism, and malnutrition, some of which were relevant in our patient's case. There is no known associated risk for actinomycosis based on race, age, or occupation [1]. The cervicofacial form of actinomycosis usually starts as a small, flat, hard swelling, under the oral mucosa or the skin of the neck, which may be painful. Following that, areas of softening develop and can evolve into sinuses and fistulas that discharge characteristic sulfur granules. The salivary glands, pharynx, tongue, cheek, cranial bones, meninges, or the brain can be affected by direct extension [4].

The diagnosis of actinomycosis usually involves culturing the organism, as in our case. Imaging could aid in providing a tentative diagnosis, but definitive diagnosis is based on culture and microscopic identification of Actinomyces spp. [1]. Typically, actinomycosis is treated with high-dose penicillin. Management of cervicofacial actinomycosis often requires prolonged courses of antibiotics, particularly due to the poor penetration of beta-lactams in the bone [5,6]. Surgical intervention may also be necessary in more complicated cases [7]. One case involving the successful treatment of thoracic actinomyces with daily intravenous ceftriaxone has been reported in the literature, suggesting that ceftriaxone can be an adequate replacement [8]. 

We presented a case with successful treatment of cervical facial actinomycosis complicated by bacteremia with intravenous ceftriaxone and oral metronidazole. We believe our findings provide deeper insights into the adequate broad-spectrum coverage of certain third- and fourth-generation cephalosporins.

Outcome and follow-up

The patient will undergo a new CT neck and CT maxillofacial, three weeks after discharge, in order to evaluate for the evolution of infection and to determine whether an extension of the antibiotic regimen would be required.

Learning points

A non-biased and comprehensive examination of these patients is important. This case highlights this by describing significant dentition findings that led to the eventual detection of Actinomyces on blood cultures and the management of the patient by oral maxillary facial surgeons for the long term.

Limitations

This report has a few limitations. Although we believe that Actinomycesinfection played a major role in the patient's clinical disease, the patient was concurrently infected with multiple other bacterial species that contributed, to an uncertain degree, to the patient's poor clinical state. Additionally, we were unable to obtain a sufficient sample from the abscess to culture bacteria to isolate the primary bacterial species, and we did not perform any histopathology studies during the hospitalization.

## Conclusions

Poor dentition is a common problem that can affect an individual's health. However, an oral exam is often overlooked in such cases. In our case, the continued anaerobic and Gram-positive coverage during the transition from intravenous vancomycin, intravenous cefepime, and oral metronidazole to intravenous ceftriaxone and oral metronidazole proved to be successful in treating a rare case of Actinomyces bacteremia from an anaerobic standpoint, even though intravenous penicillin is considered the gold standard of treatment. A multidisciplinary approach toward cervicofacial actinomycosis was critical in this patient’s treatment.

## Appendices

Patient's perspective

When the patient was told that he had a rare bacterial blood infection that would require months of treatment and follow-up, he stated “my mother always said whenever I do something, I do it big.” In general, he stated that the symptom onset was fast and aggressive, which brought him into the ED. He has been feeling “really good” since the drainage but still notes mild sore throat and voice changes. He says that he had not been to a dentist or brushed his teeth in 30 years for no particular reason. He is excited to get back to work at his auto repair shop and is thankful to have recently received insurance coverage through his job. He was worried about the long course of antibiotics, but says “I feel better knowing that I only have a few weeks of IV and then I can switch to pills.” This experience has changed his perspective on his health. He says, “I am quitting smoking and drinking… I will go to the dentist to get whatever teeth I need to get pulled.” He has also asked for a toothbrush to start brushing his teeth.
